# Nitidane: An Irregular Prenylated Diterpene from the Cuticle of the Springtail *Heteromurus nitidus*

**DOI:** 10.1021/acs.jnatprod.4c00258

**Published:** 2024-04-26

**Authors:** Anton Möllerke, Stefan Schulz

**Affiliations:** TU Braunschweig, Institute of Organic Chemistry, Hagenring 30, 38106 Braunschweig, Germany

## Abstract

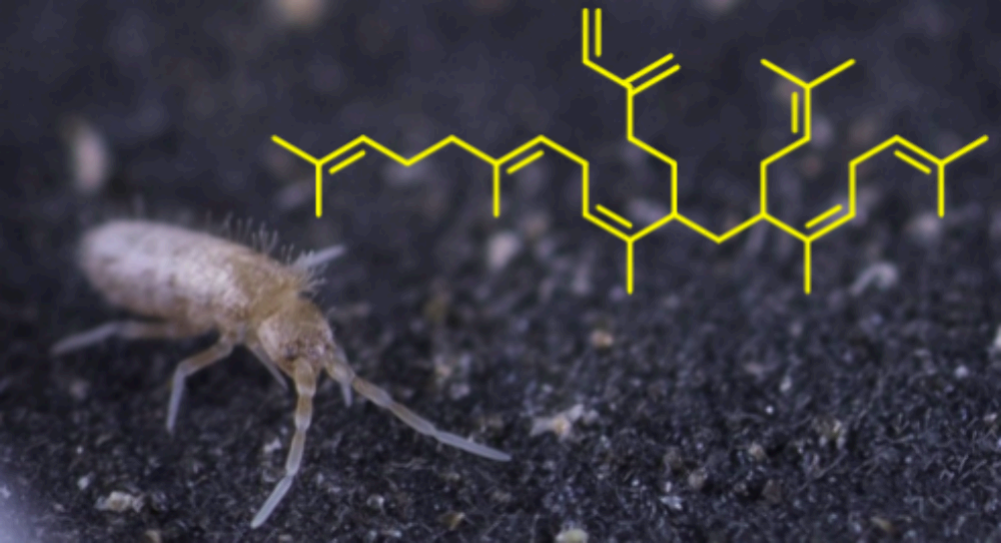

Collembola are closely related to insects, but our knowledge of their often unique chemistry is limited. Here we report the identification of the epicuticular lipid nitidane, representing a novel class of epicuticular lipids. Nitidane (**4**) is an irregular terpene consisting of seven isoprene units, made up of a diterpene core that is modified by a geranyl moiety that is itself prenylated. The observed [4^6^+(2^2^+1^1^)^1^]-terpene structure has not been reported before.

Terpene secondary metabolite biosynthesis follows a remarkably conserved biosynthetic pathway throughout all organisms. The isoprene building blocks are connected regularly head to tail up to five units to form mono-, sesqui-, di-, and sesterterpenes, while head-to-head connection finally arrives at tri- and tetraterpenes. Archaea use tail-to-tail connections, as well. Exceptions are rare, although some reports on the extension of the regular building block connection up to eight units have been published.^1−5^ Another type of connection is the formal prenylation of an open-chain terpene to arrive at branched open-chain terpenes. Examples include lavandulol (**1**), a [1^2^+1^1^]-terpene,^6^ and so-called highly branched isoprenoids (HBIs) from diatoms that are [3^6^+2^1^]- and [3^6^+3^1^]-terpenes such as **3**.^7−9^ Other compounds combining both types of the discussed unusual structural features are viaticenes A and B (**2**), [6^14^+2^1^]-terpenes from the springtail *Hypogastrura viatica*.^6^
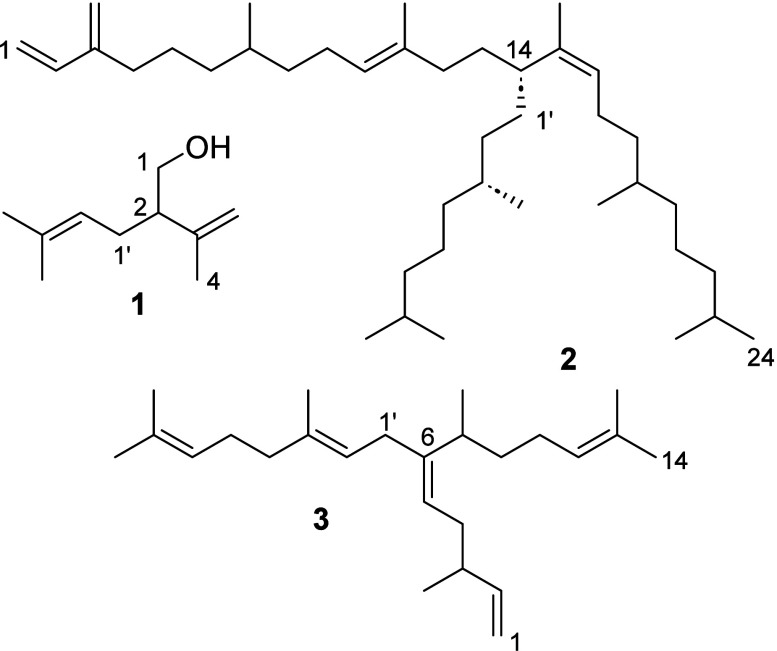


Collembola (springtails) are important soil-dwelling arthropods, a sister class to the Insecta. Although the cuticular chemistry of insects has been extensively studied,^10−12^ our knowledge of the composition of the collembolan cuticular lipids is limited to a few isolated studies.^2,6,13,14^ Insects typically rely on a complex mixture dominated by open-chain hydrocarbons, accompanied sometimes by aldehydes, esters, and other fatty acid-derived compounds.^10−12^ In contrast, the cuticle of Collembola contains a limited number of compounds, long-chain hydrocarbons, or terpenes.^2,6,15^ The cuticle of many Collembola is superhydrophobic, a characteristic that allows them to float on water, a feature not found in insects.^16−20^ This superhydrophobicity is partly attributed to the nanostructure of the cuticle, which forms a hexagonal pattern. However, this cannot explain seasonal changes in wetting behavior, where the cuticular lipids also seem to play a role.^17^ Additionally, Collembola tend to aggregate in large groups and exhibit coordinated group behavior, although the regulating mechanism is unknown.^21,22^ A mediation by contact signals such as cuticular compounds, as found in many insects, seems possible.^23^ An indication for such a role might be the presence of species-specific unique lipids, as we have described with poduran from *Podura aquatica*(2) and viaticene from *H. viatica*.^6^

We report here the identification of another unique cuticular lipid of *Heteromurus nitidus* (Entomobryidae), which we named nitidane. Nitidane extends the side chain of viaticene even further: A diterpene core structure is geranylated, but the side chain is again prenylated. The seven isoprene units are arranged in a [4^6^+(2^2^+1^1^)^1^]-terpene structure, unprecedented so far in natural products, leading to a highly branched compound.

*H. nitidus* was collected in the wild and cultured for several weeks until about 1 g of biomass was available. The Collembola were extracted with pentane. The gas chromatogram of the extract is shown in Figure 1a. In addition to the common terpenoids squalene and cholesterol, major compounds **A**, **B**, and **C** were detected. All three showed mass spectra indicating open-chain terpene compounds. The mass spectrum of **A**, the major component of the cuticular extract, is shown in Figure 1b. Because no match with known compounds in several MS databases was found, we analyzed the structure of **A** in detail.

**Figure 1:**
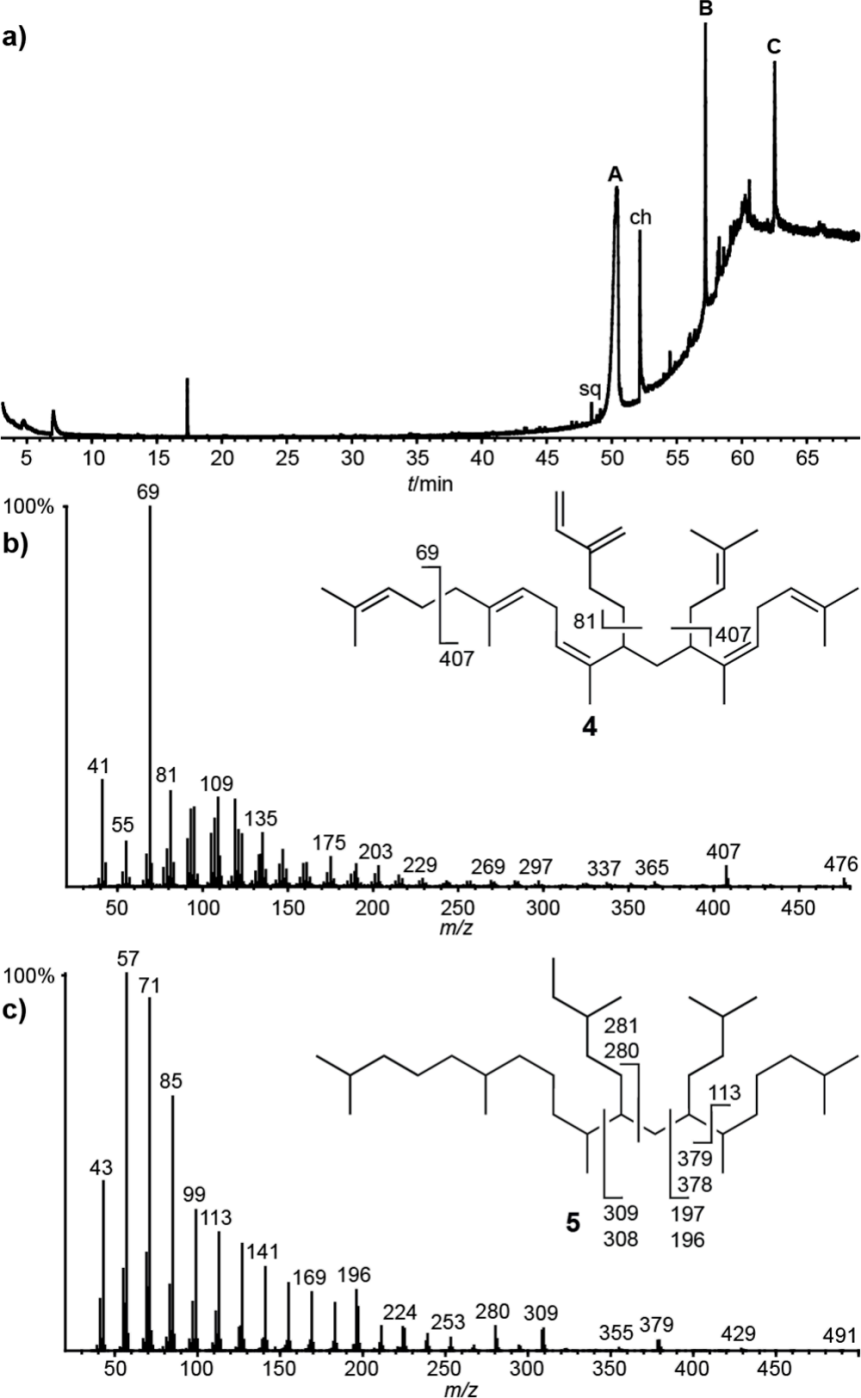
Gas chromatogram of the natural pentane extract of *H. nitidus* (a) and mass spectra of compound **A** (b) and the saturated compound **5**, the product of a microhydrogenation of **A** (c). sq = squalene; ch = cholesterol; **A**, **B**, and **C** = unknown terpenes.

High-resolution mass spectrometry (HRMS) showed a molecular ion at *m*/*z* 476.4378, indicating a molecular formula of C_35_H_56_, with eight double-bond equivalents (dbe). The fragments *m*/*z* 407 and 69 are consistent with the loss of an isoprenyl unit, indicating a terpene, as is the carbon number of 35. The ion *m*/*z* 69, often indicating a terminal dimethylallyl fragment in terpenes, was of exceptionally high intensity, hinting at the possible presence of several of these units in **A**. Compounds **B** and **C** showed similar spectra with the highest ions observed at *m*/*z* 544 and 597, consistent with one or two additional isoprene units (Figures S1, S2). Nevertheless, their concentrations were too low to allow full structure elucidation.

About 0.8 mg of compound **A** was isolated by microcolumn chromatography and analyzed by 1D and 2D NMR experiments (Figure 2, Table 1), indicating the highly branched terpene **4** to be the structure of **A**. The NMR data showed eight double bonds, of which six were trisubstituted and two were terminal, including one 1,3-butadiene motif. The 1,3-butadiene can be characterized by the correlation of the two terminal olefinic protons. The doublet of doublets at 6.36 ppm (H-2) showed HMBC correlations with C-1 and C-17, and the coupling constants indicated a neighboring proton in both *cis*- and *trans*-position (*J* = 17.5 Hz, 10.8 Hz).^24^ COSY and HMBC correlations of CH_2_-17 and CH_2_-4 show that CH_2_-4 is neighboring C-3. Further in the chain is H_2_-5, which shows COSY correlations with both H_2_-4 and H-6. Two non-olefinic CH groups with nearly identical signals (37.32, 37.13 ppm) were identified in the HSQC spectrum. We identified these CH groups as branching points CH-6 and CH-22. Key to their assignment within the chain were the HMBC correlations of CH_*2*_-21, which correlated not only with C-6 and C-22 but also with their nearest neighbors C-7 and C-23, positioning CH_2_-21 exactly between the two groups (Figure S8). The different side chains were similarly assigned. The configurations of the trisubstituted double bonds were elucidated by the NOESY correlation of the olefinic proton with the neighboring groups (Figure 2). Both double bonds adjacent to the branching points are *cis*-configured, indicated by the NOESY correlations of H_2_-29 with H-24 and H_3_-18 with H-8.^24^ This is also supported by the coupling constants of both vinylic protons *J* of 7.3 and 7.4 Hz, respectively, which is typical for *cis*-configured double bonds.^24^ The C-10–C-11 double bond is *trans*-configured, as deduced by the NOESY correlation of H_2_-12 and H-10. These data indicated that nitidane has structure **4** shown in Figure 2.

**Figure 2:**
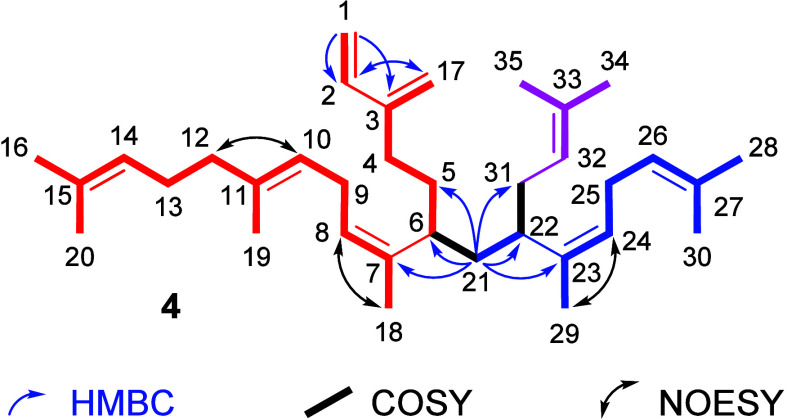
Structure of natural compound **A** with key HMBC, COSY, and NOESY correlations (^1^H = 600 MHz, ^13^C = 151 MHz, CDCl_3_). The colors identify the three terpenoid building blocks.

**Table 1:** NMR Data (^1^H 600 MHz, ^13^C 151 MHz, CDCl_3_) of Compound **A**, Nitidane (**4**)

position	^13^C [ppm], type	^1^H [ppm] (*J* [Hz])	^1^H-, ^13^C-HMBC
1	113.01, CH_2_	5.21–5.17/5.05–5.01, m	2, 3
2	139.10, CH	6.36, dd (17.5, 10.8)	1, 4, 17
3	146.95, C		1, 4
4	29.50, CH_2_	2.17–2.11/2.03–1.95, m	2, 3, 5, 17
5	31.41, CH_2_	1.60–1.53/1.48–1.40, m	4, 21
6	37.13, CH	2.59–2.54, m	7, 8, 18, 21
7	137.15, C		6, 9, 18, 21
8	125.85, CH	5.18, t (7.4)	6, 9, 18
9	26.60, CH_2_	2.78–2.73/2.71–2.66, m	7, 8, 10, 11
10	123.41, CH	5.11–5.06, m	9, 12, 19
11	134.92, C		9, 12/13,19
12	39.74, CH_2_	2.08–2.04/2.00–1.95, m	10, 11, 13, 14 19
13	26.73, CH_2_	2.09–2.03/1.99–1.96, m	11, 12, 14, 15
14	124.36, CH	5.12–5.07, m	12, 13, 16, 20
15	131.33, C		13, 16, 20
16	25.68, CH_3_	1.68, s	14, 15, 20
17	115.28, CH_2_	4.99–4.95, s	2, 4
18	18.25, CH_3_	1.56–1.58, m	6, 7, 8
19	16.08, CH_3_	1.62, s	10, 11, 12
20	17.68, CH_3_	1.60, s	14, 15, 16
21	37.83, CH_2_	1.45–1.39/1.36–1.30, m	5, 6, 7, 22, 23, 31
22	37.32, CH	2.59–2.54, m	21, 23, 24, 29, 31
23	137.37, C		21, 22, 25, 29, 31
24	125.35, CH	5.14, t (7.3)	22, 25, 29
25	26.60, CH_2_	2.73–2.69/2.65–2.63, m	23, 24, 26, 27
26	123.72, CH	5.08–5.04, m	25, 28, 30
27	131.16, C		25, 28, 30
28	25.68, CH_3_	1.68, s	26, 27, 30
29	18.35, CH_3_	1.56, s	22, 23, 24
30	17.68, CH_3_	1.61, s	26, 27, 28
31	31.66, CH2	2.10–2.04/1.96–1.88, m	21, 22, 23
32	123.34, CH	5.03–4.99, m	31, 34, 35
33	131.41, C		34, 35
34	17.88, CH_3_	1.59, s	32, 33, 35
35	25.77, CH_3_	1.66, s	32, 33, 34

Structure **4** was also supported by mass spectrometric data. The cleavage of an isoprenyl unit is the most favorable fragmentation in **4**, as two allylic cations can be formed when **4** is cleaved at C-12/13 or C-22/31. Compound **A** was also subjected to microhydrogenation with Pd/C and H_2_. This resulted in a mixture of saturated isomers **5**, presumably diastereomers with very similar mass spectra (Figure S3). One of these mass spectra is shown in Figure 1c. As is typical for long alkanes, the molecular ion is weak. In the saturated ion series, several ions show increased intensity and peak doublets (*m*/*z* 379/378, 309/308, 280, and 196), indicating at least two major branching points in **5**. The most favorable fragmentations are between the two tetrasubstituted bonds C-6/7 and C-22/23, because both secondary cations and radicals are formed. The favored fragmentation next to branching points is well-known for linear alkanes and is used, for example, to identify methyl-branched alkanes important in insect chemical ecology.^25,26^ Interestingly, in **5** the larger ions of these cleavages at *m*/*z* 379/378 and 309/308 show increased intensity, while the smaller fragments preferentially become radicals. The even-numbered ions at *m*/*z* 378 and 308 are products of hydrogen rearrangements.^27^ The increased intensity of the ions *m*/*z* 280/281 and 196/197 can be explained by cleavage at C-21/6 and C-21/22. In both cases, only the secondary cations are increased in intensity.

Attempts to clarify the configuration of the two stereogenic centers remained inconclusive. Because of the extremely flexible character of **4**, conformational calculations were unsuccessful, thus excluding DFT calculation of NMR shift values. In addition, C-6 and C-22 located at the two stereogenic centers had almost identical ^13^C NMR shift values (37.13 and 37.23 ppm). The small amount of **A** available gave an α value of only 0°. Attempts to measure UV/vis and ECD spectra were unsuccessful.

Nitidane (**4**) is the first open-chain prenylated terpene, in which the side chain is again prenylated. An open-chain diterpene core (red in Figure 2) is geranylated at C-6 (blue), which itself is prenylated at C-2 (pink), resulting in a highly branched compound. The alkylations proceed under the formation of a shifted double bond that results in a *Z*-configuration in both cases. Whether this type of double-bond formation, also observed in viaticene (**2**), is of functional importance because it leads to a more closed structure^6^ or is a requirement for proper enzyme formation remains unclear.

## Conclusion

The structure of nitidane (**4**), the major compound of the epicuticular lipids of *H. nitidus*, was established to be (5*Z*,10*Z*,13*E*)-2,6,10,14,18-pentamethyl-7-(3-methylbut-2-en-1-yl)-9-(3-methylenepent-4-en-1-yl)nonadeca-2,5,10,13,17-pentaene. Nitidane is a rare example of a terpene consisting of three irregularly connected isoprenoid precursors and underlines the unique epicuticular chemistry of Collembola. It adds to the already known collembolan irregular terpenes poduran^2^ and viaticene.^6^ As a large cuticular hydrocarbon, nitidane (**4**) helps to prevent desiccation from edaphic *H. nitidus*(28) and, due to its high abundance and structural uniqueness, may play a role in intra- and/or interspecific recognition. Furthermore, it may contribute to the superhydrophobicity of the cuticle of Collembola.

## Experimental Section

### General Experimental Procedures

Optical rotation was determined with an MCP 150 polarimeter (Anton Paar) with a cell length of 10 cm (*c* given in mg/mL). UV/vis and ECD spectra were measured on a Jasco J-720 spectropolarimeter. NMR spectra were recorded on a Bruker AVII 600 (^1^H NMR: 600 MHz, ^13^C NMR: 151 MHz) instrument. ^1^H data were referenced to the TMS signal, and ^13^C data to the residue solvent signal (CDCl_3_, 77.160 ppm). Mass spectra (EI, 70 eV) were recorded with a combination of an Agilent Technologies 5977B gas chromatograph connected to an Agilent Technologies 8860 Series MSD. Gas chromatographic retention indices were calculated against a series of *n*-alkanes according to van den Dool and Kratz^29^ using a standard HP-5MS (Agilent Technologies, 30 m, 0.25 mm i.d. 0.25 μm film thickness) phase. For HRMS an Exactive GC orbitrap mass spectrometer (ThermoScientific) was used. The resolution was set to 60,000 (fwhm; instrument setting at 200 u). Mass range was 50–650 u, and 2 microscans were averaged per data scan. Automated gain control (AGC target) was set to 1 × 10^6^, and maximum inject time was set to “auto”. Auxiliary temperatures were set to 290 °C for both transfer lines 1 and 2, and the temperature of the electron ionization source was set to 220 °C. EI was performed at a 70 eV energy in positive mode. Helium (carrier gas) and nitrogen (supply for the C-trap) lines were equipped with gas purification cartridges to trap moisture and organic impurities of the gases (Thermo Scientific). The column bleed ion at 207.03235 u was used as the lock mass for internal mass calibration of the data. For chemical ionization in positive mode (CIP), methane (99.995%) was used as the CI-gas at a flow rate of 1.5 mL/min. Column chromatography: silica 60 (0.063–0.200 mm, 70–230 mesh ASTM).

### Biological Material

A laboratory culture of *Heteromurus nitidus* was established in our laboratory. The Collembola were kept in Petri dishes. A mixture of plaster of Paris and activated charcoal (10:1) was used as the substrate. Once a month, the Collembola were placed on fresh substrate. The Collembola were fed with baker’s yeast *ad libitum*. The cultures were kept for 3 months, and about 1 g of Collembola was collected and extracted.

### Isolation of Compound **A**

The Collembola were covered with pentane (SupraSolv, Merck) in a 2 mL screw-cap glass vial. After 30 min, the extract was carefully transferred to a fresh screw-cap glass vial using a glass pipet without transfer of springtail individuals. The extract was concentrated by room-temperature evaporation and transferred onto a small-scale flash chromatography column (SiO_2_) made from a Pasteur pipet. The chromatography began with pentane (SupraSolv, Merck) as an eluate, and then dichloromethane (SupraSolv, Merck) was increasingly gradually added to the eluent. The fractions containing compound **A** were combined and concentrated (0.8 mg).

### Microhydrogenation

About 100 μL of the natural extract was transferred into a 1.5 mL vial equipped with a 200 μL insert. A minute amount of Pd/C was added to the solution. The vial was placed under a hydrogen atmosphere for 1 h using needles and hydrogen lines. The mixture was filtered through Celite, and the eluent was analyzed by GC/MS after concentration.

#### Nitidane (**4**):

colorless oil; [α]^25^_D_ 0 (0.5 mg/mL, pentane); UV (pentane) 0; ^1^H, ^13^C NMR, Table 1; EIMS *m*/*z* 476 [M]^+^ (2), 121 (15), 119 (23), 109 (23), 107 (18), 105 (15), 95 (21), 93 (21), 81 (24), 69 (100), 41 (28); HREIMS *m*/*z* 476.4378 M^+^ (calcd for C_35_H_56_, 476.4377).

## Data Availability

The raw NMR data of nitidane are added in the Supporting Information. The mass spectrum of nitidane will be included in the open-access MS database MACE after publication.^30^

## Supporting Information Available

Mass spectra, NMR spectra of nitidane (PDF)
Raw NMR data of nitidane (ZIP)
