# Supplementary material for: Nitidane: An Irregular Prenylated Diterpene from the Cuticle of the Springtail Heteromurus nitidus
Source: J Nat Prod. 2024 Apr 26;87(5):1454–8. doi: 10.1021/acs.jnatprod.4c00258 (PMC11129290; doi:10.1021/acs.jnatprod.4c00258)
Supplement: Supplementary file 1 — np4c00258_si_001.pdf [file np4c00258_si_001.pdf]

Nitidane – an Irregular Prenylated Diterpene from the Cuticle of the Springtail *Heteromurus nitidus*

Anton Möllerke, Stefan Schulz*.

TU Braunschweig, Institute of Organic Chemistry, Hagenring 30, 38106 Braunschweig, Germany.

TABLE OF CONTENTS

1. Mass Spectra 2

2. Hydrogenation Experiment 3

3. NMR Spectra 4

1. Mass Spectra

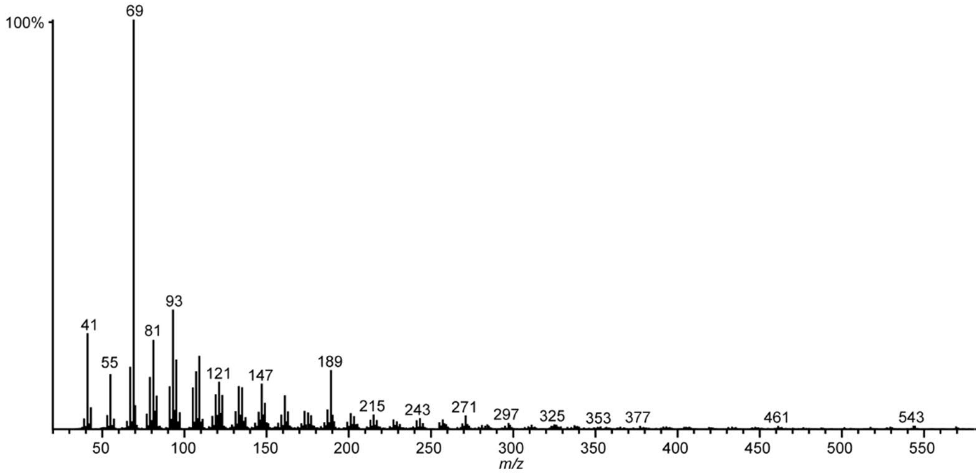

Figure S1. Mass spectrum of the natural compound **B**.

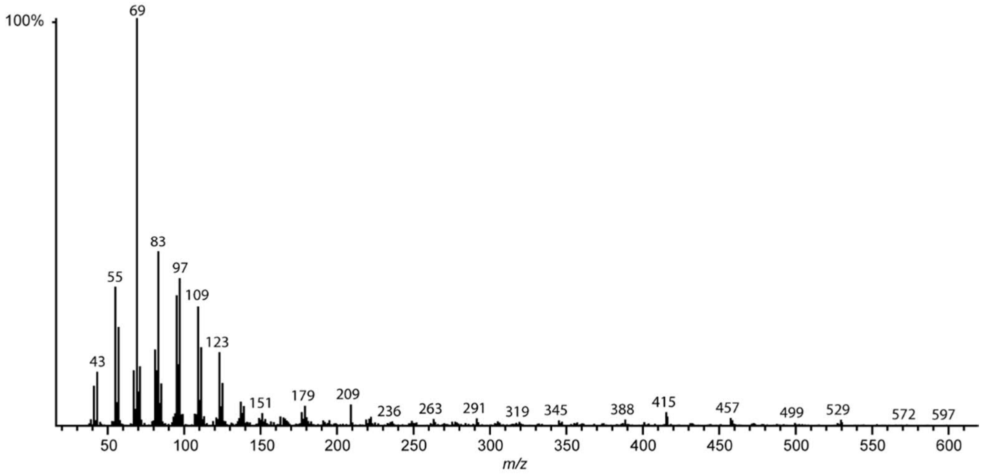

Figure S2. Mass spectrum of the natural compound **C**.

2. Hydrogenation Experiment

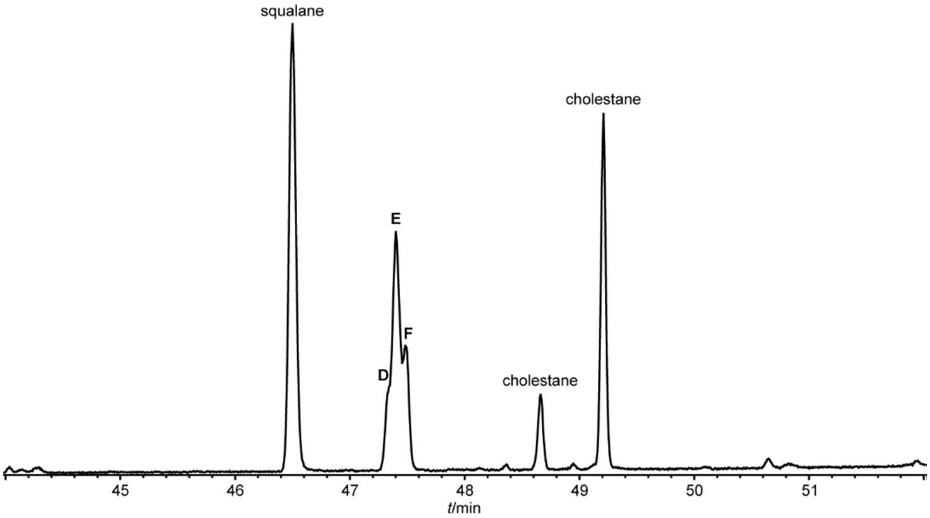

Figure S3. Gas chromatogram of the natural extract of *H. nitidus* after microhydrogenation. Compounds **D**, **E**, and **F** are the hydrogenated derivatives of **A**. The different peaks are formed due to the many stereogenic centers in the hydrogenated product.

3. NMR Spectra

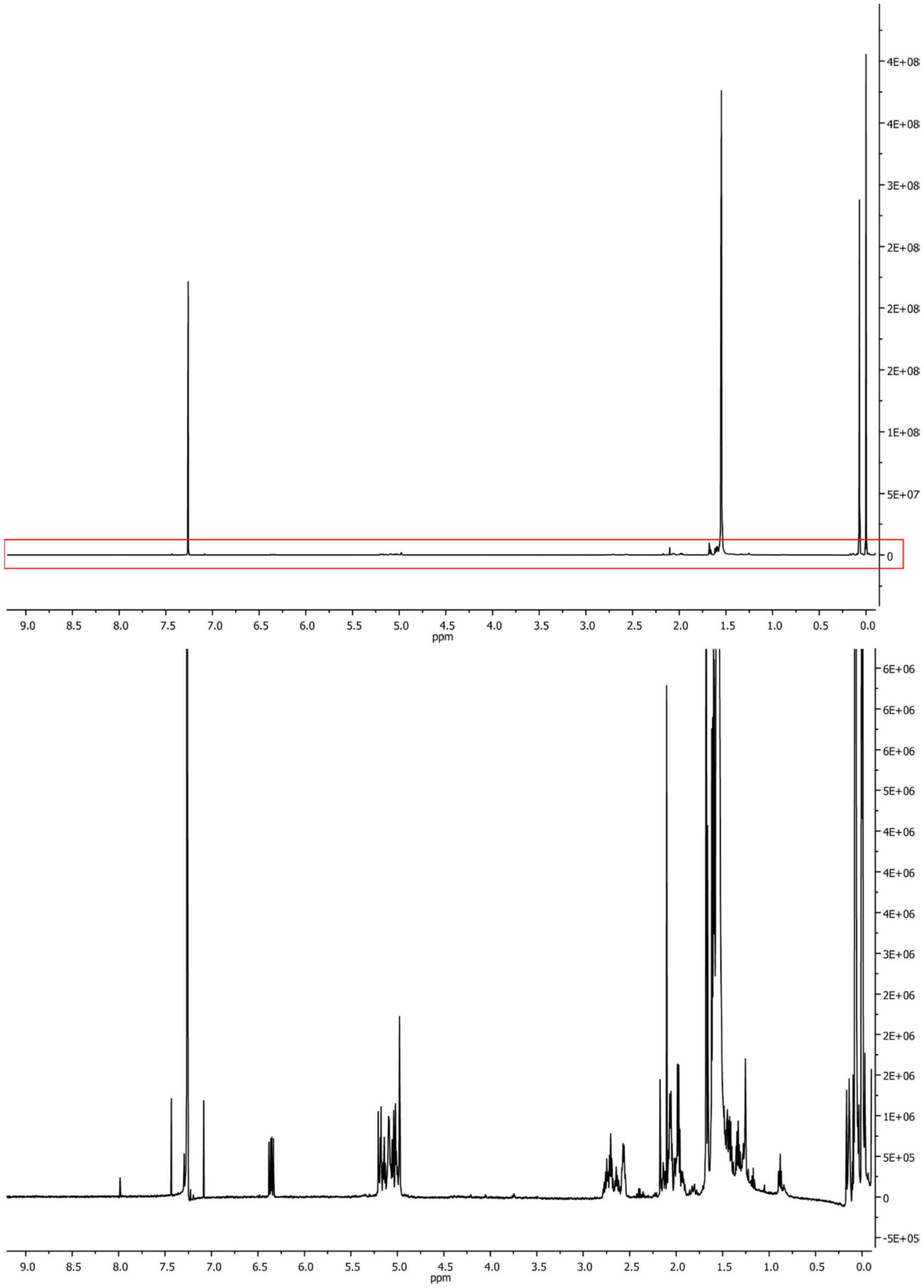

Figure S4. ^{1}H NMR spectrum ($CDCl_3$, 600 MHz) of compound **A**. Upper part: full spectrum, bottom part: detail clipping (red box).

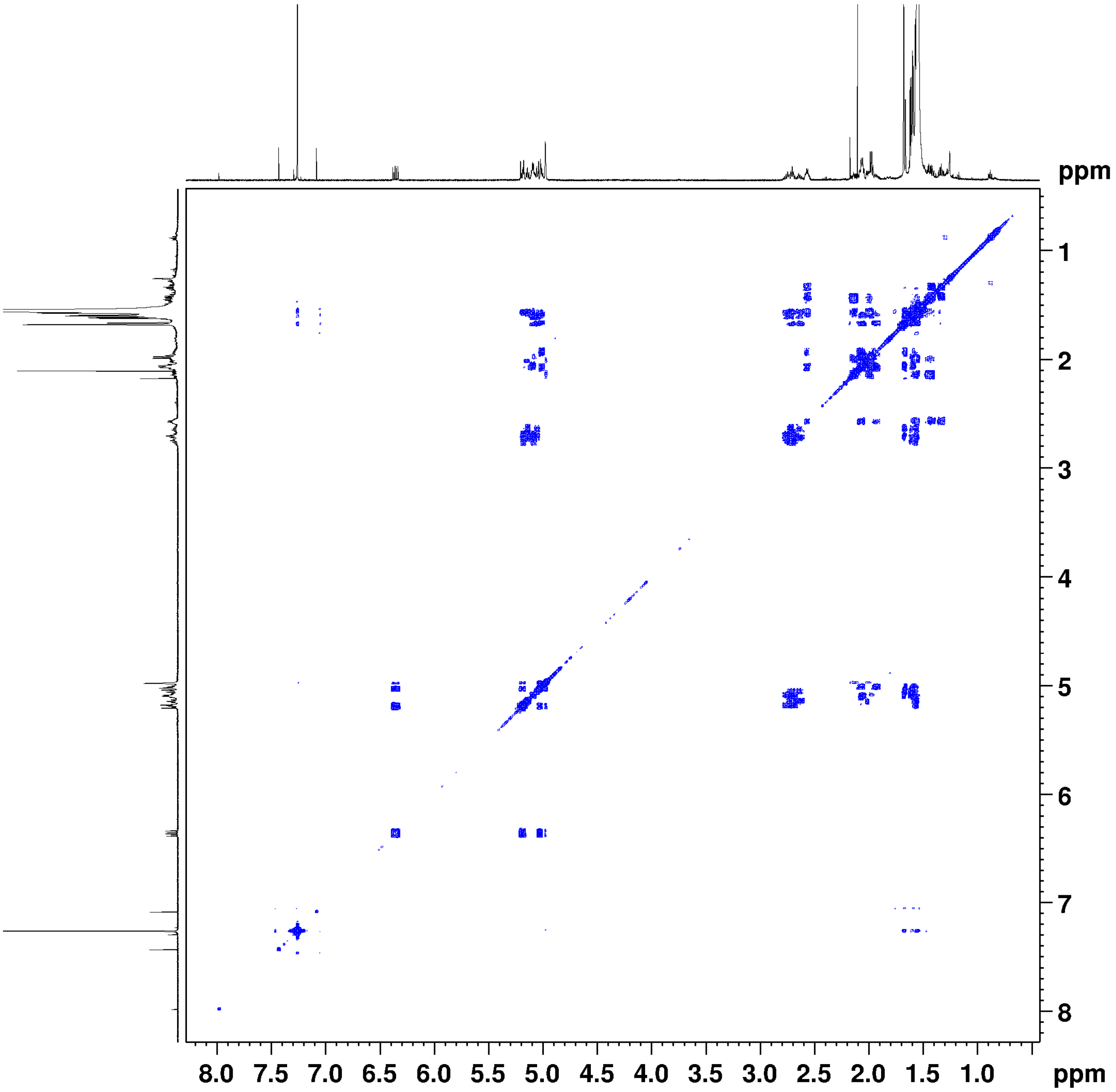

Figure S5. H,H-COSY NMR spectrum ($CDCl_3$, 600 MHz) of compound **A**.

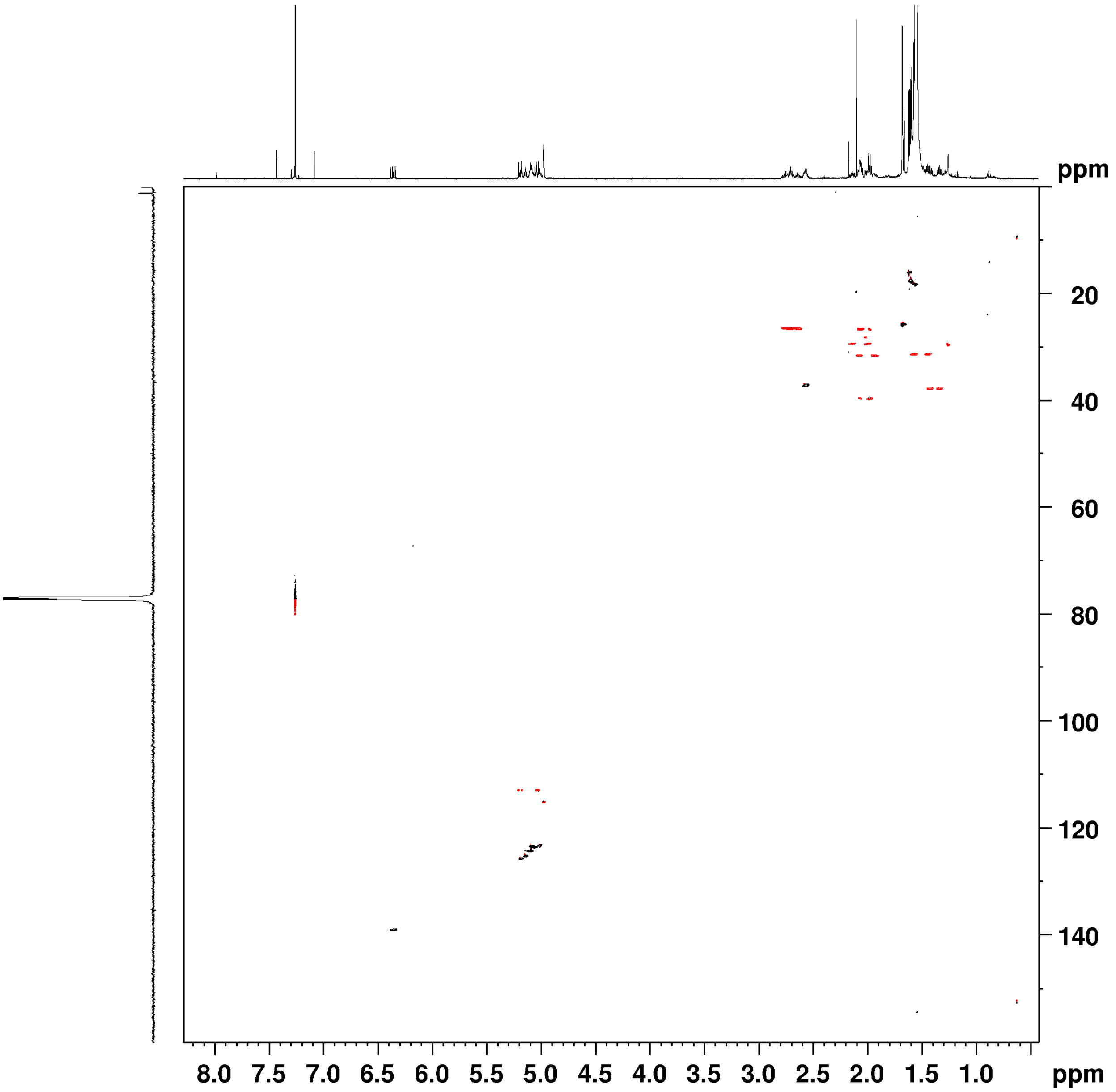

Figure S6. H,C-HSQC NMR spectrum ($CDCl_3$, ^1H 600 MHz, ^{13}C 151 MHz) of compound **A**.

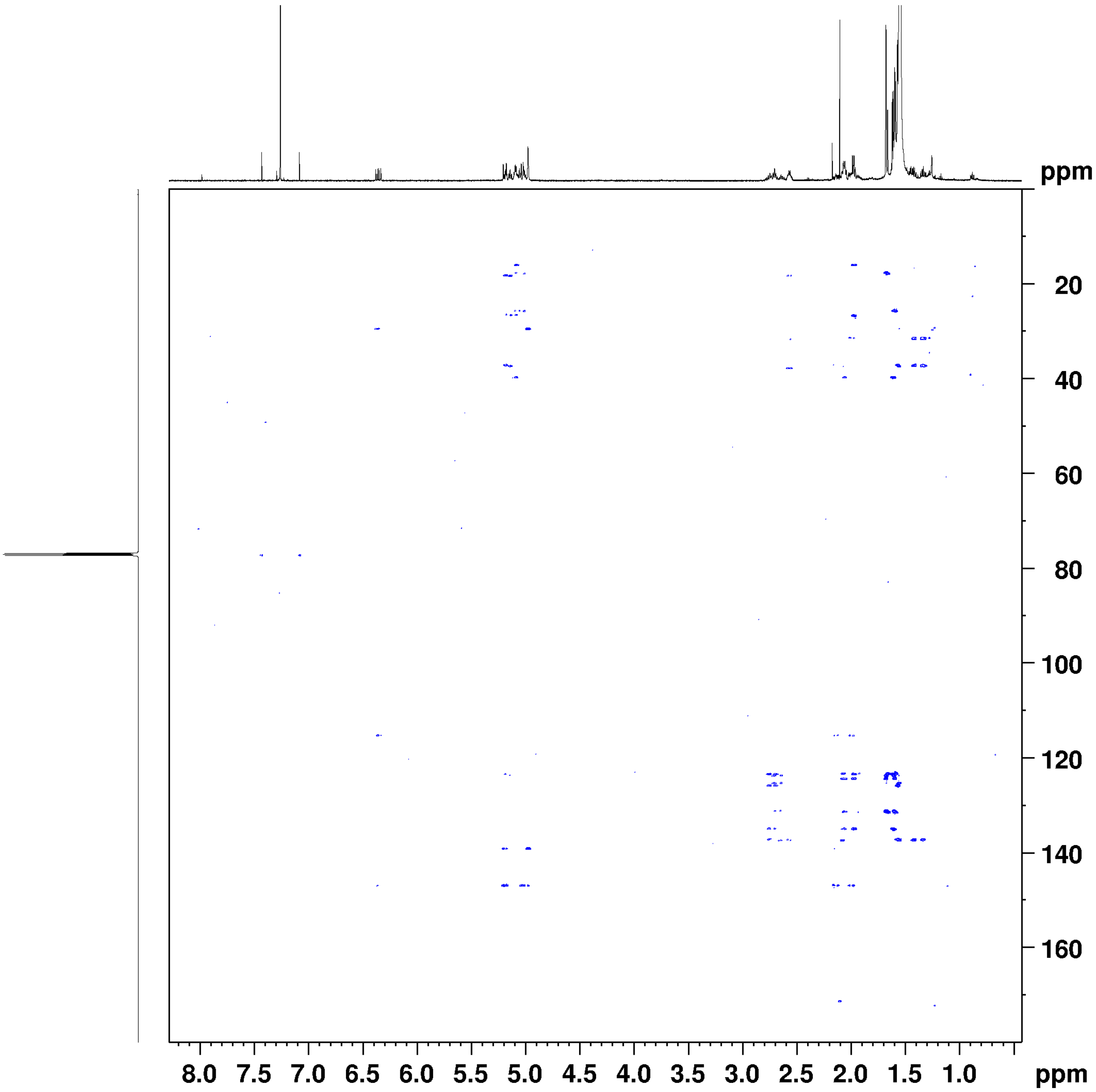

Figure S7. H,C HMBC NMR spectrum ($CDCl_3$, ^1H 600 MHz, ^{13}C 151 MHz) of compound **A**.

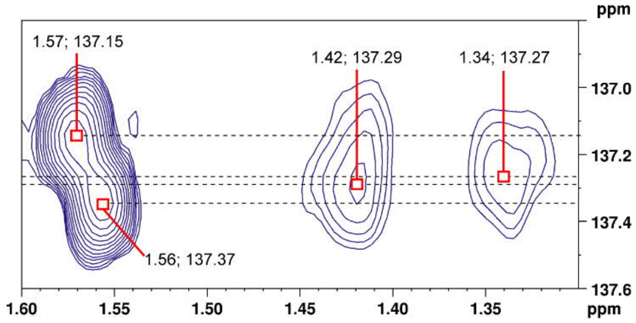

Figure S8. Highlighted key correlations of the H,C HMBC NMR spectrum ($CDCl_3$, ^1H 600 MHz, ^{13}C 151 MHz) of compound **A**. The crosspeak of H-21 (1.45-1.39/1.36-1.30 ppm) is located between C-7 (137.15 ppm) and C-23 (137.23 ppm). We therefore assigned these signals as the not resolved correlations with both C-7 and C-23.

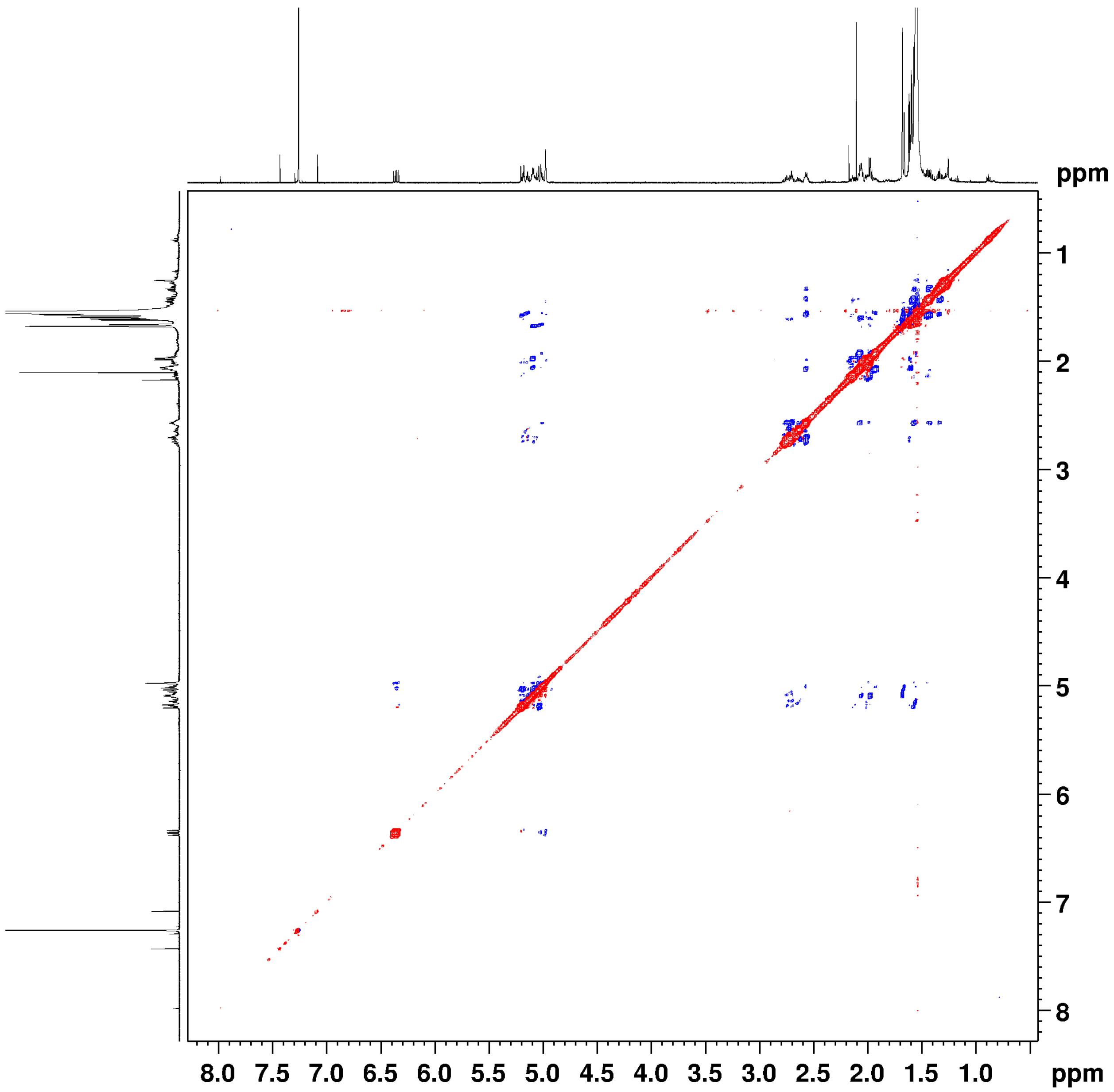

Figure S9. NOESY NMR spectrum ($CDCl_3$, 600 MHz) of compound **A**.
